# Rice DWARF14 acts as an unconventional hormone receptor for strigolactone

**DOI:** 10.1093/jxb/ery014

**Published:** 2018-01-20

**Authors:** Ruifeng Yao, Lei Wang, Yuwen Li, Li Chen, Suhua Li, Xiaoxi Du, Bing Wang, Jianbin Yan, Jiayang Li, Daoxin Xie

**Affiliations:** 1 Tsinghua-Peking Joint Center for Life Sciences, School of Life Sciences, Tsinghua University, Beijing, China; 4 MOE Key Laboratory of Bioinformatics, School of Life Sciences, Tsinghua University, Beijing, China; 2 State Key Laboratory of Plant Genomics and National Center for Plant Gene Research (Beijing), Institute of Genetics and Developmental Biology, Chinese Academy of Sciences, Beijing, China; 3 University of the Chinese Academy of Sciences, Beijing, China

**Keywords:** Arabidopsis, DWARF14, phytohormone, receptor, rice, strigolactone

## Abstract

Strigolactones (SLs) act as an important class of phytohormones to regulate plant shoot branching, and also serve as rhizosphere signals to mediate interactions of host plants with soil microbes and parasitic weeds. SL receptors in dicots, such as DWARF14 in Arabidopsis (AtD14), RMS3 in pea, and ShHTL7 in Striga, serve as unconventional receptors that hydrolyze SLs into a D-ring-derived intermediate CLIM and irreversibly bind CLIM to trigger SL signal transduction. Here, we show that D14 from the monocot rice can complement Arabidopsis *d14* mutant and interact with the SL signaling components in Arabidopsis. Our results further reveal that rice D14, similar to SL receptors in dicots, also serves as an unconventional hormone receptor that generates and irreversibly binds the active form of SLs. These findings uncover the conserved functions of D14 proteins in monocots and dicots.

## Teaser

This work reveals the conserved function of D14 from dicots and monocots, and defines rice D14 as an unconventional hormone receptor that can restore strigolactone signaling in the Arabidopsis *d14* mutant

## Introduction

Strigolactones (SLs) are a family of carotenoid-derived phytohormones (Gomez-Roldan *et al.*, 2008; Umehara *et al.*, 2008) that play a vital role in the control of plant shoot branching, a key agricultural trait that determines plant architecture and affects crop yield (Song *et al.*, 2017; Zhang *et al.*, 2017). SLs also act as rhizosphere signals to establish symbiotic interactions between plants and soil microbes (Akiyama *et al.*, 2005; Kretzschmar *et al.*, 2012; Gutjahr *et al.*, 2015), and regulate parasitic interactions by stimulating germination and growth of parasitic weeds such as Striga (Cook *et al.*, 1966; Cardoso *et al.*, 2014; Conn *et al.*, 2015; Toh *et al.*, 2015; Tsuchiya *et al.*, 2015; Gobena *et al.*, 2017; Lumba *et al.*, 2017*a*). Moreover, SLs also regulate hypocotyl elongation, root growth, leaf development and senescence (Snowden *et al.*, 2005; Kapulnik *et al.*, 2011; Ruyter-Spira *et al.*, 2011; Waters *et al.*, 2012*b*; Waters and Smith, 2013; Yamada *et al.*, 2014; Soundappan *et al.*, 2015; Ueda and Kusaba, 2015; Wang *et al.*, 2015; Li *et al.*, 2016). Recently, SLs were revealed to regulate various plant stress responses including drought tolerance and disease resistance (Umehara *et al.*, 2010; Dor *et al.*, 2011; Kohlen *et al.*, 2011; Bu *et al.*, 2014; Ha *et al.*, 2014; Decker *et al.*, 2017).

Genetic and molecular characterization of highly branched mutants in various plant species, such as rice *dwarf14* (*d14*), *d3*, *d10*, *d17*, *d27*, and *d53* (Ishikawa *et al.*, 2005; Arite et al., 2007, 2009; Gao *et al.*, 2009; Lin *et al.*, 2009; Liu *et al.*, 2009; Jiang *et al.*, 2013; Zhou *et al.*, 2013), Arabidopsis *more axillary growth1* (*max1*), *max2*, *max3*, *max4*, and *d14* (Woo *et al.*, 2001; Stirnberg *et al.*, 2002; Sorefan *et al.*, 2003; Booker *et al.*, 2004, 2005; Shen *et al.*, 2007; Nelson *et al.*, 2011; Waters *et al.*, 2012*a*, *b*; Abe *et al.*, 2014; Chevalier *et al.*, 2014; Yao *et al.*, 2016), pea *ramosus1* (*rms1*), *rms2*, *rms3*, *rms4*, and *rms5* (Beveridge *et al.*, 1996; Morris *et al.*, 2001; Foo *et al.*, 2005; Johnson *et al.*, 2006; de Saint Germain *et al.*, 2016), and petunia *decreased apical dominance 1* (*dad1*), *dad2*, and *dad3* (Snowden *et al.*, 2005; Simons *et al.*, 2007; Hamiaux *et al.*, 2012), suggests that SL biosynthesis and signaling pathway are largely conserved in diverse plant species.

Rice *D3* or its ortholog in Arabidopsis (*MAX2*), petunia (*PhMAX2A*), or pea (*RMS4*) encodes an F-box protein (Johnson *et al.*, 2006; Shen *et al.*, 2007; Stirnberg *et al.*, 2007; Drummond *et al.*, 2011; Hamiaux *et al.*, 2012), a subunit of SCF (Skp1–Cullin1–F-box protein) ubiquitin ligase complex that functions in substrate recognition for proteasome-mediated proteolysis. Rice *D14* or its orthologs, such as Arabidopsis *D14,* petunia *DAD2*, or pea *RMS3*, encodes an α/β hydrolase that hydrolyzes SLs (Hamiaux *et al.*, 2012; Nakamura *et al.*, 2013; Zhao *et al.*, 2013; de Saint Germain *et al.*, 2016; Yao *et al.*, 2016) and interacts with its respective F-box protein in an SL-dependent manner to recruit various repressors, such as D53 in rice and SMXL6/7/8 (SUPPRESSOR OF MAX2 1-LIKE6/7/8) proteins in Arabidopsis, for ubiquitination and degradation (Hamiaux *et al.*, 2012; Jiang *et al.*, 2013; Zhou *et al.*, 2013; Soundappan *et al.*, 2015; Wang *et al.*, 2015; Liang *et al.*, 2016; Yao *et al.*, 2016). Degradation of these repressors will subsequently de-repress their directly targeted transcription factors, such as IPA1 (Ideal Plant Architecture 1) in rice (Song *et al.*, 2017), to activate downstream genes essential for various SL-regulated plant responses (Lumba *et al.*, 2017*b*; Waters *et al.*, 2017). It is intriguing that the receptors AtD14 and D14 are also degraded in an SL-induced and MAX2/D3-dependent manner (Chevalier *et al.*, 2014; Hu *et al.*, 2017).

Recently, AtD14 was defined as a non-canonical hormone receptor that possesses dual functions: AtD14 acts as an enzyme to hydrolyze SLs and generate the active form of the hormone molecule CLIM (the covalently linked intermediate molecule), and also serves as a receptor of SL to bind CLIM irreversibly and undergo significant conformational change for interacting with downstream components and triggering SL signal transduction (Snowden and Janssen, 2016; Yao *et al.*, 2016; Fang and Chen, 2017; Yao et al., 2017). Similarly to AtD14, RMS3 in pea and ShHTL7 in *Striga hermonthica* also hydrolyze strigolactone into the D-ring-derived intermediate CLIM and covalently bind CLIM in an irreversible manner to trigger SL signaling (de Saint Germain *et al.*, 2016; Yao *et al.*, 2017).

Rice is an important crop and also serves as a model plant for the study of monocots. It is known that rice, but not Arabidopsis, is the host plant for both symbiotic arbuscular mycorrhizal fungi (AMF) and parasitic Striga (Yoshida and Shirasu, 2009; Waters *et al.*, 2017). In this study, we employ bioinformatics, genetic, and biochemical approaches to investigate functional conservations between rice D14 and Arabidopsis AtD14, and examine whether the rice D14 is similar to AtD14 in generating and perceiving the active form of SLs.

## Materials and methods

### Generation of transgenic plants

The modified binary vector pCAMBIA1300-cFlag (Yao *et al.*, 2016) carrying the full coding sequence of *Arabidopsis thaliana D14* (*AtD14*), *Oryza sativa D14*, or N-terminus (amino acids 1–51) truncated *O. sativa D14* (*D14ΔN*) under the control of the 35S promoter was introduced into the *Atd14-5* mutant (Yao *et al.*, 2016) by using the *Agrobacterium*-mediated floral dip method. Similarly, the pCAMBIA1300-cFlag vector carrying *D14ΔN* under the control of the *AtD14* promoter was introduced into the *Atd14-1* mutant (Waters *et al.*, 2012*b*) to generate transgenic plants, *AtD14pro:D14ΔN*. The primary rosette branching numbers were counted for 7-week-old plants which were germinated on plates and grown in soil under a light/dark photoperiod of 16 h/8 h at 22 °C.

### Real-time PCR (RT-PCR) analysis

Rosette leaves of plants were collected for RNA extraction. Total RNA was prepared with a *TransZol* Kit (TransGen) and used in the reverse transcription reaction with the reagent TransScript^®^ RT/RI Enzyme Mix (TransGen). The first-strand cDNA was used as the template for the subsequent RT-PCR, which was performed to amplify *AtD14* (primers 5'-ATGAGTCAACACAACATCTTAG-3' and 5'-GATGATTCCGATCATAGCG-3'), and *D14* (primers 5'-TGACCTCTTCGCCAAGCTTG-3' and 5'-TCTTGAAGACG GTCTGGCAGAC-3') in the plants with the indicated genotypes. The *A. thaliana ACTIN1* was employed as a control (primers 5'-TGTTGAGAAGAACTACGAGC-3' and 5'- AAGCACTTCCTGTGAACAAT-3').

### Hypocotyl measurements

The Arabidopsis seeds were sterilized and germinated on Murashige and Skoog (MS) medium with or without 3 μM *rac*-GR24 (an SL analog, Chiralix) under continuous low light at 22 °C for 7 d. Hypocotyl length was measured by Digimizer software.

### Leaf morphology analysis

Plants were grown in soil under a light/dark photoperiod of 16 h/8 h at 22 °C for an additional 3 weeks after germination and growth on MS medium for 1 week. For each genotype, 20 plants were used for observation of leaf morphology. Whole plants and their sixth leaves were photographed and harvested for further measurement. The leaf length (the distance between the leaf tip and the base of petiole) and leaf width (the greatest distance across the leaf lamina perpendicular to the proximal/distal axis of the leaf) were measured manually using a ruler.

### Protein preparation

N-terminus-truncated *O. sativa* D14ΔN or full-length *A. thaliana* D14 (AtD14) was expressed in *Escherichia coli* strain BL21 (DE3) (Novagen) as an N-terminal glutathione *S*-transferase (GST) tag-fusion protein. After being purified by glutathione Sepharose 4B (GE Healthcare) affinity chromatography, GST–D14ΔN or GST–AtD14 protein was released by 10 mM glutathione (GSH) elution or on-column cleavage to remove the GST tag, then further purified by HiTrap Q (GE Healthcare) followed by Superdex 200 10/300 (GE Healthcare) in a buffer containing 10 mM Tris–HCl, pH 8.0, 150 mM NaCl, and 5 mM DTT.

Full-length *A. thaliana* SMXL6 was expressed in sf9 insect cells with an N-terminal Flag tag and purified by anti-Flag beads (Sigma, A2220) according to the manufacturer’s manual.

The full-length *O. sativa* D3 or *A. thaliana* MAX2 was fused with His_6_ and co-expressed with ASK1, which stabilizes F-box proteins (Yan *et al.*, 2013; Yao *et al.*, 2016; Li et al., 2017), in sf9 insect cells. After purification by Ni-NTA (Novagen) affinity chromatography, the His-D3–ASK1 or His-MAX2–ASK1 complex was eluted and further purified by HiTrap Q followed by Superdex 200 10/300 in a buffer containing 20 mM MES, pH 6.5, 150 mM NaCl, and 5 mM DTT.

### Pull-down assay

For the interaction between D14 and MAX2, ~20 μg of His-MAX2–ASK1 was used as the bait and ~12 μg of GST–D14 was used as the prey in the presence of 20 μM *rac*-GR24 or its solvent DMSO as the control. The reaction mixtures were incubated with Ni-NTA beads (Qiagen) at 4 °C for 1 h in the reaction buffer [50 mM Tris–HCl, pH 6.8, 100 mM NaCl, 25 mM imidazole, 10% (v/v) glycerol, 0.1% Tween-20, 20 mM 2-mercaptoethanol]. After washing six times with the reaction buffer, the protein complexes on the beads were released and then subjected to western blot analysis. The pull-down assay of AtD14 with MAX2 was performed in a similar way to serve as a positive control.

For the interaction between D14 and SMXL6, ~20 μg of Flag-SMXL6 protein was used as the bait and ~12 μg of GST–D14 as the prey in the presence of 20 μM *rac*-GR24 or its solvent DMSO as the control. The reaction mixtures were incubated with anti-flag beads at 4 °C for 1 h in the reaction buffer (50 mM Tris–HCl, pH 7.0, 150 mM NaCl, 0.5% Tween-20). After washing six times with the reaction buffer, the protein complexes on the beads were released and then subjected to western blot analysis. The pull-down assay of AtD14 with SMXL6 was performed in a similar way to serve as a positive control.

GST-fused proteins were detected by a monoclonal anti-GST antibody (Abmart). The polyvinylidene difluoride (PVDF) membrane was stained with Memstain (Applygen) to show equal loading.

### Co-immunoprecipitation (Co-IP) assay in protoplasts

Protoplasts prepared from the *smxl6 smxl7 smxl8* triple mutant or the wild type were transformed with transient expression plasmids as described (Wang *et al.*, 2015). After transformation with the hemagglutinin (HA)-AtD14 and green fluorescent protein (GFP)–MAX2 plasmids and incubation at 21 °C for 11 h, protoplasts were pre-treated with 100 µM *rac*-GR24 for 1 h in W5 solution. Cells were then collected and broken in the protein extraction buffer [50 mM Tris–HCl, 150 mM NaCl, 10% (v/v) glycerol, 0.1% Nonidet P-40, and 1×complete protease inhibitor cocktail], and immunoprecipitation (IP) with agarose-conjugated anti-GFP monoclonal antibody (MBL) was subsequently carried out in the presence or absence of 100 µM *rac*-GR24 at 4 °C. The HA-AtD14 recombinant proteins were detected with the anti-HA monoclonal antibody (Millipore), and the GFP–MAX2 fusion proteins and GFP were detected with the anti-GFP monoclonal antibody (Sigma). The total proteins extracted from protoplasts before IP were used as inputs.

### Size exclusion chromatography (SEC) assay

Purified D14 (~10 μM) and D3–ASK1 proteins (~5 μM) were incubated with 200 μM 5-deoxystrigol (5DS; OlChemIm Ltd) or an equal amount of DMSO as the solvent control at 25 °C for 1 h in buffer containing 20 mM MES, pH 6.5, 150 mM NaCl, 5 mM DTT. The reaction mixture was then injected onto a Superdex 200 10/300 column for analysis at a flow rate of 0.3 ml min^–1^. The fractions (0.5 ml per fraction) were analyzed by SDS–PAGE and visualized by Coomassie Brilliant Blue staining.

### Mass spectrometric analysis of covalent modification

The gel bands of D14 from the SEC-separated D14–D3–ASK1 complex induced by 5DS were excised for mass spectrometric analysis as previously described (Yao *et al.*, 2016). Briefly, tandem mass spectrometry (MS/MS) spectra from each LC-MS/MS run were searched against the D14 protein database using the Proteome Discoverer (Version 1.4) searching algorithm. The search criteria were as follows: full enzymatic specificity for trypsin was required, two missed cleavages were allowed, carbamidomethylation was set as a fixed modification (on the cysteine residue), oxidation (on the methionine residue) was set as a variable modification, precursor ion mass tolerance was 10 ppm for all mass spectra acquired in the Orbitrap mass analyzer, and fragment ion mass tolerance was 0.02 Da for all MS/MS spectra acquired in the ion trap. A high confidence score filter [false discovery rate (FDR) <1%) was used to select the ‘hit’ peptides, and their corresponding MS/MS spectra were manually inspected.

## Results

### Phylogenetic analysis and sequence alignment of D14 orthologs from monocots and dicots

**Fig. 1.**
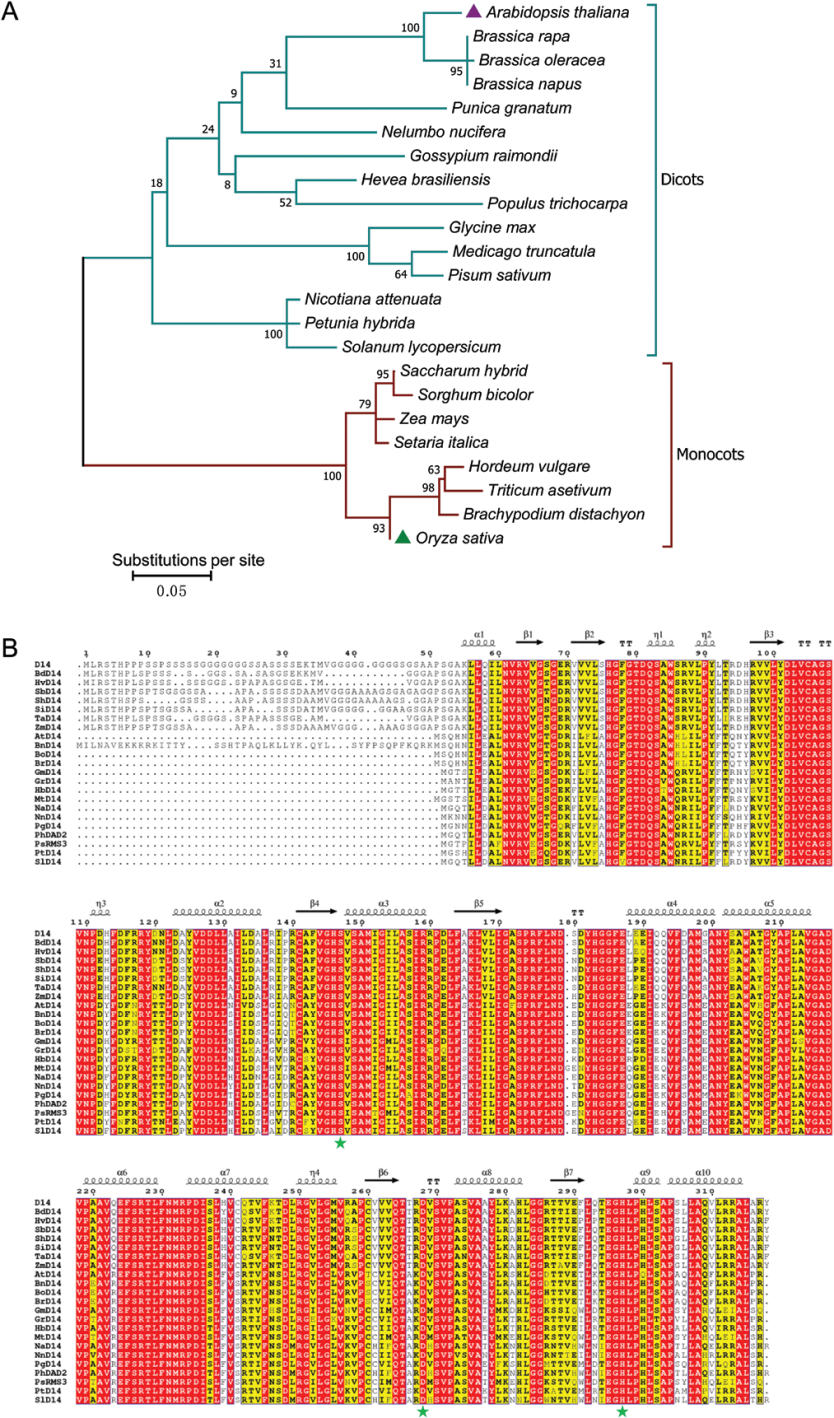
Phylogenetic analysis and sequence alignment of D14 orthologs from monocots and dicots. (A) Phylogenetic analysis of D14 orthologs from monocots and dicots. The phylogenetic tree was generated with 23 full-length amino acid sequences of D14 orthologs using the Maximum Likelihood method based on the WAG model (100 replicates) in MEGA7 (Whelan and Goldman, 2001; Kumar *et al.*, 2016). The percentage of trees in which the associated taxa clustered together is shown next to the branches. The tree is drawn to scale, with branch lengths measured in the number of substitutions per site. The GenBank accession numbers of D14 orthologs in the presented species are, from top to bottom: *Arabidopsis thaliana* D14 (NP_566220), *Brassica rapa* D14 (XP_009130408), *Brassica oleracea* D14 (XP_013638430), *Brassica napus* D14 (CDY42894), *Punica granatum* D14 (OWM70752), *Nelumbo nucifera* D14 (XP_010248100), *Gossypium raimondii* D14 (XP_012451974), *Hevea brasiliensis* D14 (XP_021646820), *Populus trichocarpa* D14 (XP_002302409), *Glycine max* D14 (XP_003557012), *Medicago truncatula* D14 (XP_003589086), *Pisum sativum* RMS3 (AMB61024), *Nicotiana attenuata* D14 (XP_019258478), *Petunia hybrida* DAD2 (AFR68698), *Solanum lycopersicum* D14 (XP_004238093), *Saccharum* hybrid D14 (AJY78078), *Sorghum bicolor* D14 (XP_002468316), *Zea mays* D14 (NP_001150635), *Setaria italica* D14 (XP_004985292), *Hordeum vulgare* D14 (AJP07999), *Triticum asetivum* D14 (AK332360), *Brachypodium distachyon* D14 (XP_003558555), and *Oryza sativa* D14 (XP_015631400). (B) Sequence alignment and structural annotation of D14 orthologs. ESPript was used to analyze the multiple sequence alignments generated by Clustal Omega (Sievers *et al.*, 2011; Robert and Gouet, 2014) with the D14 orthologs listed in (A). Secondary structure elements of the rice D14 crystal structure (PDB code: 4IH9) are displayed on top of the alignments. Identical and conserved residues are highlighted by red and yellow backgrounds, respectively. The three catalytic residues, Ser, Asp, and His, are indicated by green stars.

To investigate the evolutionary relationships among D14 orthologs in monocots and dicots, we searched public sequence databases using BLAST with the rice (*O. sativa*) D14 protein sequence as a query to obtain the predicted sequences of D14 orthologs from important monocots and dicots (Waters *et al.*, 2012*b*; Conn *et al.*, 2015; Bythell-Douglas *et al.*, 2017) (Fig. 1A). The phylogenetic analysis showed that D14 proteins from the monocots such as *O. sativa*, *Brachypodium distachyon*, *Triticum asetivum*, *Hordeum vulgare*, *Setaria italica*, *Zea mays*, *Sorghum bicolor*, and *Saccharum* hybrid have closer phylogenic relationships, while D14 orthologs from all the tested dicots exhibit closer relationships (Fig. 1A).

Further sequence alignment and structural annotation showed that the examined D14 orthologs from various plant species all exhibit considerable identities at the amino acid level and have the same catalytic triad Ser–His–Asp and α/β hydrolase fold (Fig. 1B; Supplementary Table S1 at *JXB* online), suggesting the conserved physiological functions for different D14 proteins. Consistent with phylogenetic analysis (Fig. 1A), protein sequences of D14 orthologs from monocots or dicots, respectively, are more conserved. For example, the rice D14 exhibits no less than 80% identity with its orthologs from all the tested monocots, but displays only ~50% identity with those from the examined dicots (Supplementary Table S1). Interestingly, all the examined monocot D14 proteins (including rice D14) have an additional glycine- and serine-rich N-terminus (at least 7 glycine and 10 serine residues among the N-terminal 55 residues), which is absent in all the examined dicot D14 proteins (Fig. 1B). It is unclear whether full-length D14 proteins in monocots and dicots might have some divergent physiological functions due to their discrepant N-termini.

### Rice D14 rescues phenotypes of the Arabidopsis *d14* mutant and interacts with Arabidopsis MAX2 and SMXL6 proteins

We further investigated whether the physiological function of D14 proteins is conserved in the monocot rice and the dicot Arabidopsis. We generated the transgenic Arabidopsis *35Spro:D14* and *35Spro:D14ΔN* by introducing full-length rice *D14* or N-terminus (residues 1–51) truncated rice *D14* (*D14ΔN*) under the control of the *Cauliflower mosaic virus* (CaMV) 35S promoter into the Arabidopsis *Atd14-5* mutant, a weak allele of the *Atd14* mutant (Yao *et al.*, 2016). We also introduced the 35S promoter-driven *AtD14* into *Atd14-5* to generate the *35Spro:AtD14* plants for comparison. As shown in Supplementary Table S2, 66.1% of *35Spro:AtD14* plants (39 out of 59 transgenic lines) and 33.9% of *35Spro:D14ΔN* plants (21 out of 62 lines) display a similar branching phenotype to the wild-type Col-0 (with ≤3 branches) while only 3 out of 52 (5.8%) *35Spro:D14* transgenic lines rescues the branching phenotype of *Atd14-5* well. These genetic complementation results demonstrate that the highly branched phenotype of the Arabidopsis *Atd14-5* mutant can be rescued by both the full-length rice *D14* and the N-terminus-truncated rice *D14* (*D14ΔN*), but the complementation ratio is very low for the case of full-length rice *D14*.

**Fig. 2.**
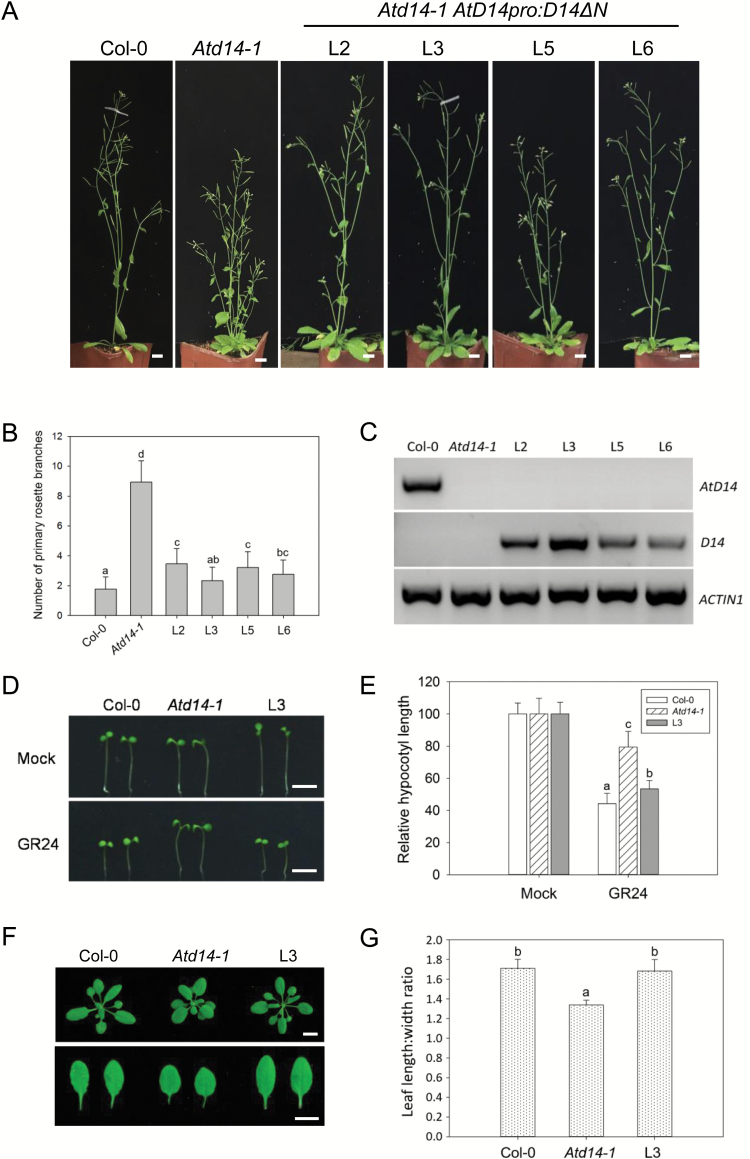
Rice *D14* rescues the Arabidopsis *d14* mutant. (A and B) Rice *D14* rescued the branching phenotype of *Atd14-1*. (A) Representative branching phenotypes of 7-week-old Col-0, *Atd14-1*, and four independent T_3_ transgenic lines *AtD14pro:D14ΔN* L2 (line 2), L3, L5, and L6 in the *Atd14-1* background; scale bars=1 cm. (B) Quantitative analysis of primary rosette branches of the indicated plants; data are means ±SD (*n*=20). Error bars indicate the SD; bars with the same letter are not significantly different from one another (ANOVA+Tukey HSD, *P*<0.01). (C) RT–PCR analysis of the *AtD14* or *D14* transcript levels in the indicated plants described in (A). The Arabidopsis *ACTIN1* was used as an internal control. (D and E) Rice *D14* rescued the hypocotyl phenotype of *Atd14-1*. (D) Representative hypocotyl phenotypes of 7-day-old Col-0, *Atd14-1*, and *AtD14pro:D14ΔN* L3 (T_3_) seedlings; scale bars=5 mm. (E) Relative hypocotyl lengths of the indicated seedlings; data are means ±SD (*n*=30). Error bars indicate the SD; bars with the same letter are not significantly different from one another (ANOVA+Tukey HSD, *P*<0.01). (F and G) Rice *D14* rescued the leaf phenotype of *Atd14-1*. (F) Representative leaf phenotypes of 4-week-old Col-0, *Atd14-1*, and *AtD14pro:D14ΔN* L3 (T_3_); scale bars=1 cm. (G) Quantitative analysis on the leaf length/leaf width ratio for the sixth leaves of the indicated plants; data are means ±SD (*n*=20). Error bars indicate the SD; bars with the same letter are not significantly different from one another (ANOVA+Tukey HSD, *P*<0.01).

We further introduced *D14ΔN* under the control of the native *AtD14* promoter into the T-DNA insertion knockout mutant *Atd14-1* (Waters *et al.*, 2012*b*) to generate the *AtD14pro:D14ΔN* plants. The results showed that 50% of the *AtD14pro:D14ΔN* plants exhibit a similar branching phenotype to the wild-type Col-0 (Fig. 2A, B; Supplementary Table S2), demonstrating that the highly branched phenotype of the Arabidopsis *Atd14-1* mutant can be well rescued by rice *D14ΔN* (Fig. 2A–C).

Moreover, we explored whether rice *D14ΔN* is able to complement *Atd14-1* in other SL-regulated physiological phenotypes including hypocotyl elongation (Scaffidi *et al.*, 2014; Umehara *et al.*, 2015) and leaf morphology (Waters *et al.*, 2012*b*). As shown in Fig. 2D–G, the *Atd14-1* mutant shows impaired sensitivity to *rac*-GR24 treatment on hypocotyl inhibition and has rounder and broader leaves than the wild-type Col-0, which is consistent with previous observations (Waters *et al.*, 2012*b*; Scaffidi *et al.*, 2014; Umehara *et al.*, 2015). However, the hypocotyl elongation of *AtD14pro:D14ΔN* plants (L3) was obviously inhibited when treated with *rac*-GR24, and the leaf phenotypes of *AtD14pro:D14ΔN* plants (L3) were also similar to those of Col-0 (Fig. 2D–G). These results demonstrate that rice *D14* can rescue many SL-regulated physiological phenotypes of the Arabidopsis *d14* mutant well.

**Fig. 3.**
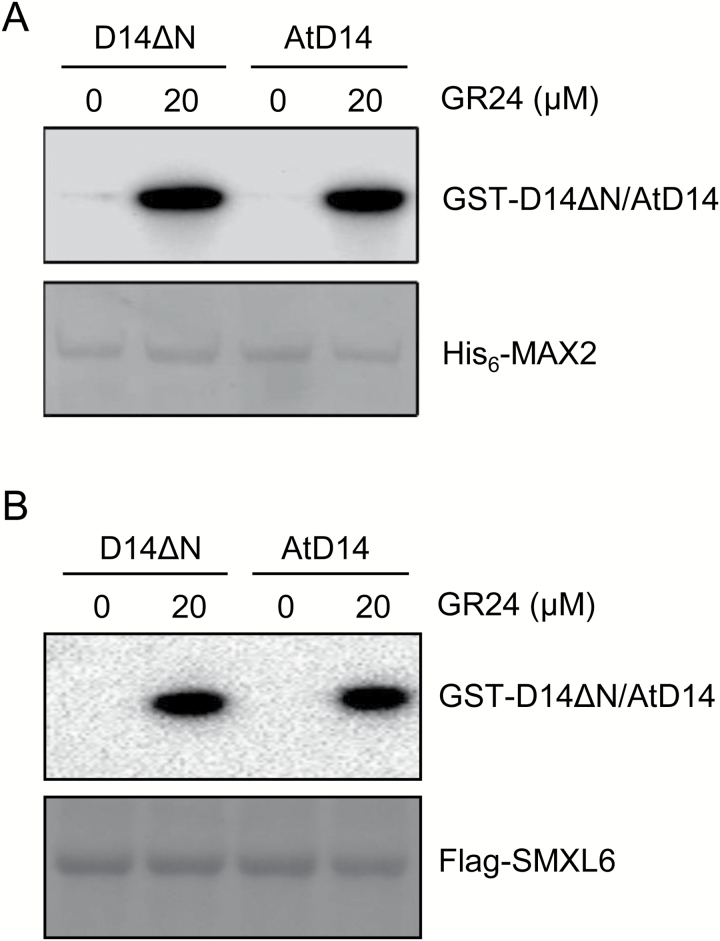
Rice D14 physically interacts with the Arabidopsis SL signaling components. (A) Rice D14 efficiently bound Arabidopsis MAX2 in the presence of *rac*-GR24. Pull-down assay using recombinant His_6_-MAX2 and GST–D14 or GST–AtD14 in the absence or presence of *rac*-GR24. GST-fused proteins were detected by anti-GST antibody and the PVDF membrane was stained with MemStain to show equal loading. (B) Rice D14 efficiently bound Arabidopsis SMXL6 in the presence of *rac*-GR24. Pull-down assay using recombinant Flag-SMXL6 and GST–D14 or GST–AtD14 in the absence or presence of *rac*-GR24. GST-fused proteins were detected by anti-GST antibody and the PVDF membrane was stained with MemStain to show equal loading.

Consistent with the functional complementation of *Atd14* by rice D14 and D14ΔN, biochemical pull-down assays and structural analysis showed that, similar to AtD14, both rice D14 and D14ΔN are able to interact efficiently with Arabidopsis MAX2 and SMXL6 proteins in an SL-dependent manner (Fig. 3; Supplementary Fig. S1). These results reveal the molecular basis of the functional complementation by showing the conserved functions of D14 proteins at the protein–protein interaction level.

Taken together, our results demonstrate that rice D14 can rescue the phenotype of the Arabidopsis *d14* mutant well, which is probably attributed to the conserved function of rice D14 to interact with Arabidopsis MAX2 and SMXL6 proteins in the presence of SLs.

### The *in vivo* interaction of the SL receptor with its F-box protein does not require repressors

**Fig. 4.**
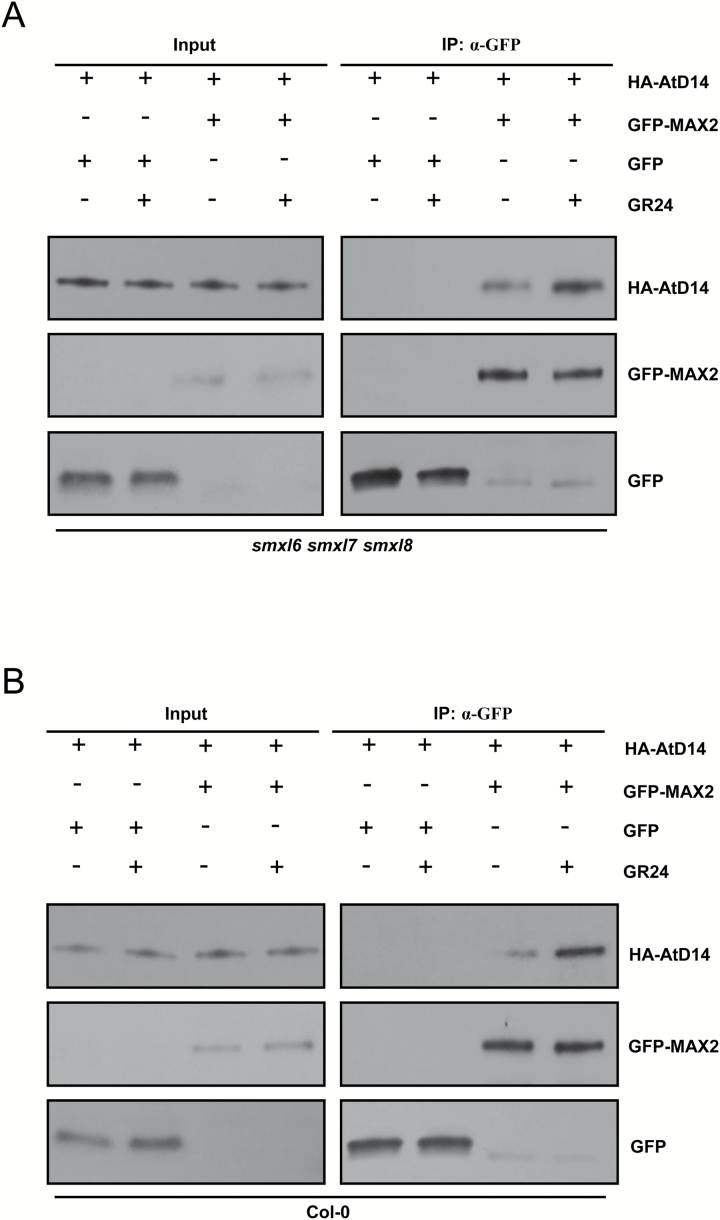
AtD14 interacts with MAX2 independently of SMXL6/7/8. The *in vivo* interactions between HA-AtD14 and GFP–MAX2 revealed by co-immunoprecipitation (Co-IP) assay in protoplasts prepared from the *smxl6 smxl7 smxl8* triple mutant (A) and the wild-type Col-0 (B). After transformation and incubation for 11 h, protoplasts were pre-treated with *rac*-GR24 for 1 h, cells were broken, and then immunoprecipitation (IP) using agarose-conjugated anti-GFP monoclonal antibody was carried out in the presence or absence of 100 µM *rac*-GR24. The HA-AtD14 recombinant protein was detected by anti-HA monoclonal antibody, and the GFP–MAX2 fusion protein and GFP were detected by anti-GFP monoclonal antibody. Input means extracted crude proteins without immunoprecipitation.

The interaction dynamics or sequential binding among SL signaling components remains an open question (Wang and Smith, 2016). It is known that the SL-induced interaction of receptor with repressor does not depend on the F-box protein MAX2 *in vitro* or *in vivo* (Wang *et al.*, 2015). However, it is unclear whether or not the SL-induced *in vivo* interaction of the SL receptor with its F-box protein is independent of repressors. To answer this question, we employed our well-established Arabidopsis protoplasts transformation and Co-IP system to investigate the interaction between AtD14 and MAX2 in the wild-type Col-0 and the *smxl6 smxl7 smxl8* triple mutant. We found that AtD14 was able to interact weakly with MAX2 in both the *smxl6 smxl7 smxl8* triple mutant (Fig. 4A) and the wild-type Col-0 (Fig. 4B), and that such interactions in both the triple mutant and Col-0 were obviously enhanced by the addition of exogenous *rac*-GR24 (Fig. 4). These data demonstrate that the SL-induced *in vivo* interaction of AtD14 with MAX2 is independent of SMXLs. Together with previous studies (Wang *et al.*, 2015), these results imply that the *in vivo* interactions between any two components among the receptor (AtD14 or D14), the F-box protein (MAX2 or D3), and the repressor (SMXLs or D53) do not require the presence of the third one.

### Rice D14 is an unconventional hormone receptor for SLs

**Fig. 5.**
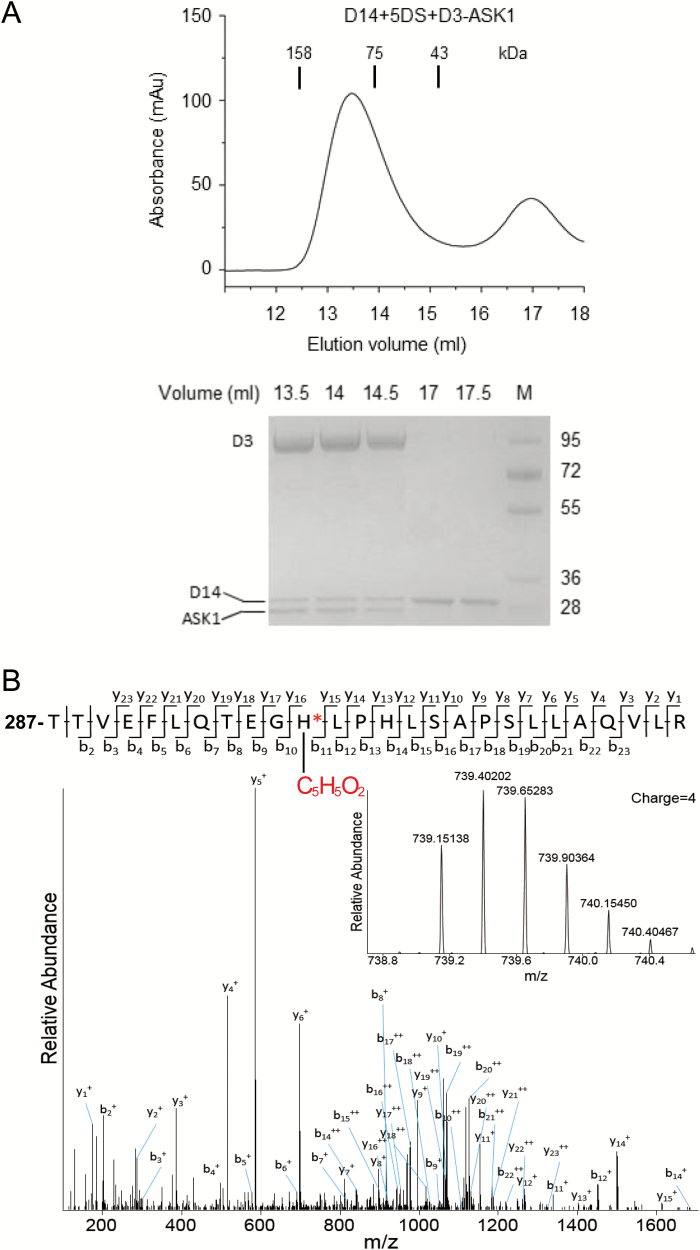
Rice D14 generates and covalently binds the active form of SLs. (A) 5DS induced the interaction of rice D14 and D3 in the SEC assay. Upper panel: SEC analysis of the interaction between D14 and D3–ASK1 in the presence of 5DS; the elution volumes of the molecular weight markers are indicated above the peaks. Lower panel: SDS–PAGE analysis of peak fractions from the upper panel; M, molecular weight ruler (kDa). (B) Rice D14 hydrolyzed 5DS and generated the C_5_H_5_O_2_ modification on the catalytic residue H297. A quadruply charged peptide (287-TTVEFLQTEGHLPHLSAPSLLAQVLR-312) of D14 with the 5DS-derived C_5_H_5_O_2_ modification on H297 was identified by MS/MS (*m/z*=739.40202). The modified peptide was isolated from the trypsin digestion products of D14 in the 5DS-induced D14–D3–ASK1 complex collected in SEC (A). Labeled peaks correspond to masses of y and b ions of the peptide displayed on the top, respectively. The asterisked ‘H’ indicates the modified H297.

To investigate further whether rice D14, similar to AtD14, generates and covalently binds the active SL molecule CLIM, we employed the SEC approach to prepare the 5DS-induced D14–D3–ASK1 complex for MS/MS analysis. The D14–D3–ASK1 complex was eluted earlier (fraction peak ~13.4 ml) (Fig. 5A, upper panel), and then subjected to SDS–PAGE to separate D14 protein (Fig. 5A, lower panel) for further trypsin digestion followed by MS/MS analysis. Peptide matching from MS/MS spectra identified a chemically modified peptide (287-TTVEFLQTEGHLPHLSAPSLLAQVLR-312) of D14 with a molecular weight shift of 96.0211 Da on the catalytic residue H297 (Fig. 5B), which is identical to the accessional molecular weight on the corresponding histidine residue (H247) of AtD14 (Yao *et al.*, 2016). As the control, no modified peptide was identified when D14 without 5DS treatment was subjected to MS/MS analysis (Supplementary Fig. S2).

**Fig. 6.**
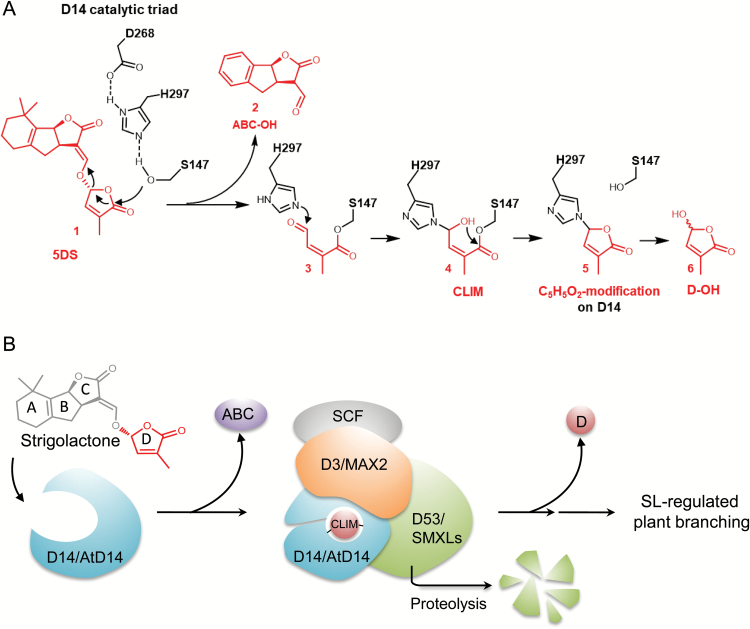
Proposed mechanism of SL perception by rice D14. (A) Schematic diagram of a proposed rice D14-mediated hydrolysis process of 5DS. The hydrolysis of 5DS (**1**) is proposed to involve a nucleophilic attack by S147, which produces ABC-OH (**2**) and compound **3**, and the generation of D-OH (**6**). The N^ε2^ atom of H297 attacks the aldehyde carbon atom of the S147-linked compound **3** to form the H297- and S147-linked linear compound **4**, referred to as the covalently linked intermediate molecule (CLIM). Compound **4** initiates an intramolecular nucleophilic attack to generate the H297-linked circular compound **5**, which appears as a C_5_H_5_O_2_ modification on H297 detected by MS/MS and denotes the existence of CLIM inside the D3-bound D14. Compound **5** would be further hydrolyzed from H297 to produce D-OH (**6**). A similar deduction of the AtD14-mediated (+)-GR24 hydrolysis process can be found in detail in our recent study (Yao *et al.*, 2016). (B) A simplified model of SL perception. D14/AtD14 docks SL in the catalytic cavity, hydrolyzes SL into a D-ring-derived intermediate (CLIM), which is covalently sealed inside the catalytic center of D14/AtD14 to promote the interaction with the D3/MAX2-based SCF complex and the repressor D53/SMXLs for triggering SL-regulated plant branching (Yao *et al.*, 2016).

Taking advantage of the comprehensive analyses on D14-mediated SL perception in our recent work (Yao *et al.*, 2016), we are able to deduce that this molecular weight shift of 96.0211 Da (Fig. 5B) corresponds to the chemical formula C_5_H_4_O_2_, which indicates covalent C_5_H_5_O_2_ modification on H297 of D14 (compound 5, Fig. 6A) and denotes the presence of D-ring-derived intermediate compound 4 (CLIM; Fig. 6A) as the active form of SLs in the SL-induced D14–D3 complex. Moreover, the same C_5_H_5_O_2_ modification on rice D14 was also detected *in planta* when *35Spro:D14ΔN* plants were treated with 5DS (Supplementary Fig. S3).

Together with previous studies about D14 function in rice (Arite *et al.*, 2009; Gao *et al.*, 2009; Liu *et al.*, 2009; Jiang *et al.*, 2013; Zhou *et al.*, 2013), our results collectively uncover the conserved function of D14 proteins in the monocot rice and the dicot Arabidopsis, and suggest that rice D14 acts as an unconventional hormone receptor to generate and perceive the active form of SLs.

## Discussion

Understanding of hormone perception is central to comprehending hormone action. Biologists over the past century have established a general perception mechanism for phytohormones: receptors specifically and reversibly bind their ligands with high affinity to initiate hormone signaling, and eventually release the unchanged ligands for the next round of perception. However, recent works on SL perception in dicots (de Saint Germain *et al.*, 2016; Yao *et al.*, 2016, 2017) have defined Arabidopsis D14, pea RMS3, and Striga HTL7 as unconventional receptors that hydrolyze SLs into the active form of hormone (CLIM), covalently bind CLIM to trigger SL signaling, and ultimately release an inactive hydrolysis product D-OH. Here, our data suggest that D14 in the monocot rice possesses the same physiological functions as AtD14, and also acts as an unconventional hormone receptor to generate and perceive CLIM, and expectedly undergo conformational changes (Hamiaux *et al.*, 2012; Zhao *et al.*, 2015; Yao *et al.*, 2016) for recruitment of signaling components (such as D3 and D53) (Jiang *et al.*, 2013; Zhou *et al.*, 2013), thereby triggering SL signal transduction (Fig. 6B).

It is intriguing that a glycine- and serine-rich N-terminus is present in all the examined monocot D14s but absent in those of all the tested dicots (Fig. 1), and the N-terminus-truncated rice D14 showed a much higher complementation ratio than the full-length rice D14 when expressed in the Arabidopsis mutant *Atd14-5* (Supplementary Table S2). However, the underlying molecular mechanism remains to be investigated in the future. Such a glycine- and serine-rich N-terminus does not affect the D14 interaction with MAX2 or SMXL6 (Supplementary Fig. S1), and is possibly structurally ﬂexible (Kagiyama *et al.*, 2013). Given that D14 can be transported via phloem in rice (Kameoka *et al.*, 2016), it would be interesting to investigate whether the additional glycine- and serine-rich N-terminal sequence of rice D14 functions as a signal peptide for D14 localization and/or transport *in vivo*.

Divergent features of the SL signaling pathway in monocotyledonous and dicotyledonous species are also found in the downstream signal transduction process. In the dicots Arabidopsis and pea, the gene *BRANCHED1* (*BRC1*), which encodes a TCP transcription factor, has been demonstrated to be a key SL-responsive gene in the downstream SL signaling pathway (Aguilar-Martínez *et al.*, 2007; Póza-Carrion *et al*., 2007; Mashiguchi *et al.*, 2009; González-Grandío *et al.*, 2013; Wang *et al.*, 2015). However, in the monocots rice and maize, the *BRC1* ortholog *TEOSINTE BRANCHED1* (*TB1*) was not up-regulated by GR24 treatment (Minakuchi *et al.*, 2010; Guan *et al.*, 2012). Moreover, a recent study identified rice IPA1 (OsSPL14), a member of the SQUAMOSA PROMOTER BINDING PROTEIN-LIKE (SPL) transcription factor family (Jiao *et al.*, 2010; Miura *et al.*, 2010), as a direct target of the repressor D53 to participate in the SL-mediated regulation of rice tillering (Song *et al.*, 2017). The loss-of-function mutant of *IPA1* is insensitive to GR24 treatment and shows more tillers than wild-type rice plants (Song *et al.*, 2017). Similar regulation of *Triticum aestivum* (Ta)D53 on TaSPL13/17 from bread wheat was also observed recently (Liu *et al.*, 2017). However, the Arabidopsis mutant containing loss-of-function mutations in both *SPL9* and *SPL15* (the orthologs of the *IPA1*/*OsSPL14* gene) still responds to the SL analog GR24 and shows reduced branching to a level similar to that of the wild type (Bennett *et al.*, 2016). These phenomena suggest that the downstream SL signaling pathway seems not to be fully conserved between monocots and dicots. Further study is needed to better understand the elusive downstream SL signaling pathway in various plant species.

## Supplementary data

Supplementary data are available at *JXB* online.

Fig. S1. The N-terminus of rice D14 does not affect the interaction with Arabidopsis components MAX2 or SMXL6. 

Fig. S2. No modified peptide was identified when D14 without 5DS treatment was subjected to MS/MS analysis.

Fig. S3. SL can generate the C_5_H_5_O_2_ modification of rice D14 *in planta*. 

Table S1. Protein identities between D14 orthologs and D14 or AtD14.

Table S2. Branch numbers of *Atd14* transgenic plants with full-length or truncated rice *D14*.

Supplementary Tables S1-S2 and Figures S1-S3

## Abbreviations:

BRC: BRANCHED
CLIM: covalently linked intermediate molecule
D: DWARF
DAD: DECREASED APICAL DOMINANCE
GFP: green fluorescent protein
IP: immunoprecipitation
IPA: Ideal Plant Architecture
MAX: MORE AXILLARY GROWTH
MS/MS: tandem mass spectrometry
RMS: RAMOSUS
SCF: Skp–Cullin–F-box
SEC: size exclusion chromatography
SL: strigolactone
SMXL: SUPPRESSOR OF MAX2 1-LIKE
SPL: SQUAMOSA PROMOTER BINDING PROTEIN-LIKE
TB: TEOSINTE BRANCHED.
