# Supplementary material for: Rice DWARF14 acts as an unconventional hormone receptor for strigolactone
Source: J Exp Bot. 2018 Jan 20;69(9):2355–65. doi: 10.1093/jxb/ery014 (PMC5913607; doi:10.1093/jxb/ery014)
Supplement: Supplementary Tables S1-S2 and Figures S1-S3 [file ery014_suppl_supplementary_tables_s1-s2_and_figures_s1-s3.pdf]

D14 orthologs in different species	*Oryza sativa*	*Arabidopsis thaliana*
Oryza sativa	-	52%
Brachypodium distachyon	**86%**	54%
Hordeum vulgare	**84%**	54%
Sorghum bicolor	**80%**	55%
Saccharum hybrid	**81%**	55%
Setaria italica	**84%**	55%
Triticum asetivum	**82%**	54%
Zea mays	**82%**	54%
Arabidopsis thaliana	52%	-
Brassica napus	52%	**78%**
Brassica oleracea	54%	**90%**
Brassica rapa	54%	**91%**
Glycine max	51%	**62%**
Gossypium raimondii	52%	**70%**
Hevea brasiliensis	52%	**68%**
Medicago truncatula	49%	**65%**
Nicotiana attenuata	54%	**70%**
Nelumbo nucifera	52%	**70%**
Punica granatum	53%	**72%**
Petunia hybrida	54%	**70%**
Pisum sativum	49%	**63%**
Populus trichocarpa	50%	**67%**
Solanum lycopersicum	54%	**68%**

Table S1. Protein identities between D14 orthologs and D14 or AtD14.

Construct	Background	Number of hygromycin-resistant T1 lines	Number of T1 lines with branches≤3	Ratio
Empty Vector	*Atd14-5*	60	0	0
35Spro:AtD14	*Atd14-5*	59	39	66.1%
35Spro:D14	*Atd14-5*	52	3	5.8%
35Spro:D14ΔN	*Atd14-5*	62	21	33.9%
AtD14pro:D14ΔN	*Atd14-1*	30	15	50.0%

Branches number of control plants: Col-0: 2.1±0.6 (n=30); *Atd14-5*: 6.4±1.0 (n=30); *Atd14-1*: 12.2±1.0 (n=30).

Table S2. Branch numbers of *Atd14* transgenic with full-length or truncated rice *D14*.

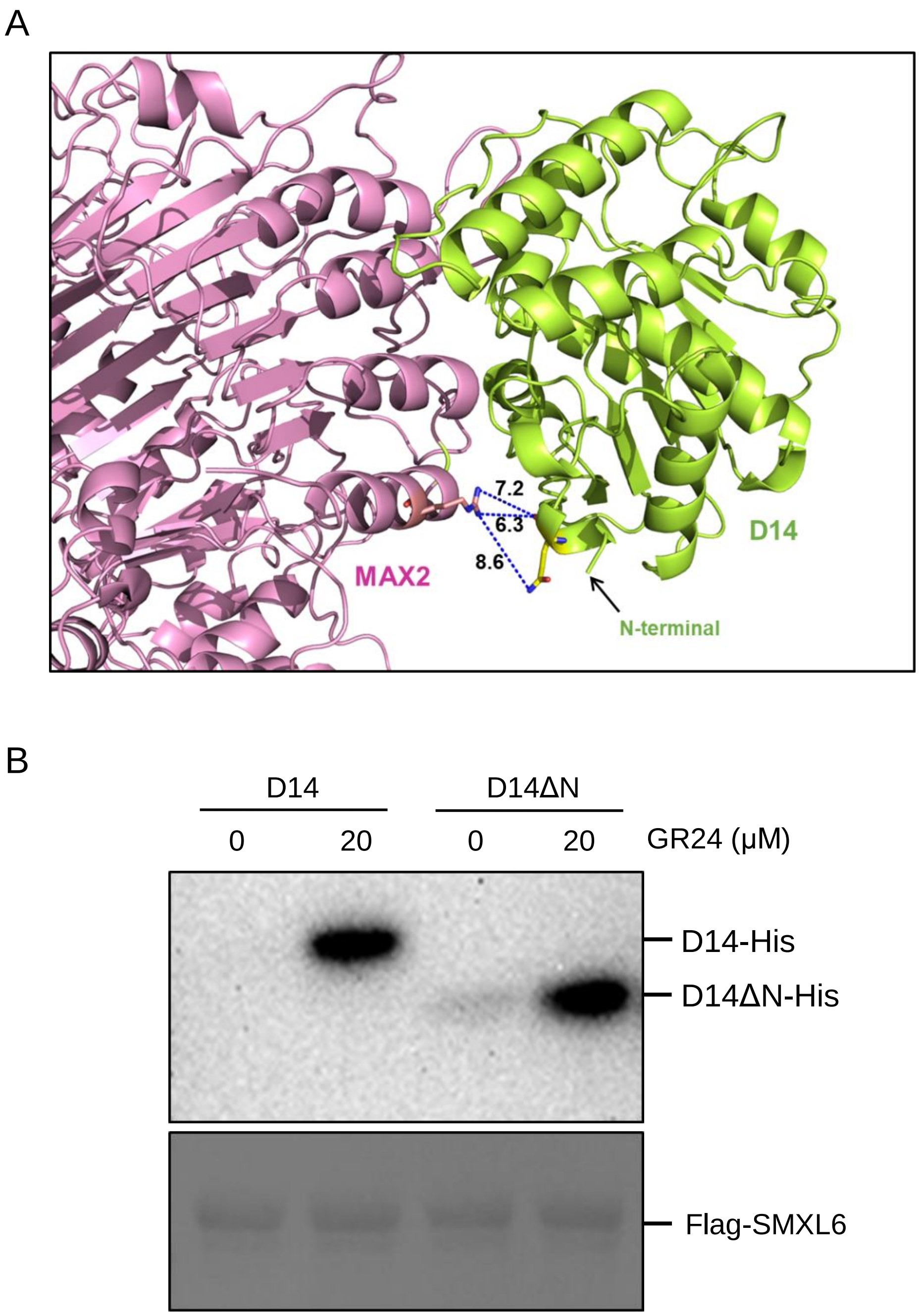

Fig. S1. The N-terminal of rice D14 does not affect the interaction with Arabidopsis components MAX2 or SMXL6.

(A) The N-terminal of rice D14 would not affect the interaction with MAX2. A homology model based on AtD14 and D3 from the CLIM-AtD14-D3-ASK1 complex (PDB code: 5HZG) for the rice D14 and Arabidopsis MAX2 complex is shown as cartoon representation and coloured as limon and pink, respectively. The closest distant between MAX2 and the N-terminal of rice D14 is farther than 6.3 Å, which suggests that the N-terminal of rice D14 does not participate in the interaction with MAX2.

(B) The N-terminal of rice D14 does not affect the interaction with SMXL6. Pull-down assay using recombinant Flag-SMXL6 and D14-His or D14ΔN -His in the absence or presence of *rac*-GR24. D14 and D14ΔN showed similar ability to bind Arabidopsis SMXL6 in the presence of *rac*-GR24. His-tag-fused proteins were detected by anti-His antibody and the PVDF membrane was stained with Memstain to show equal loading.

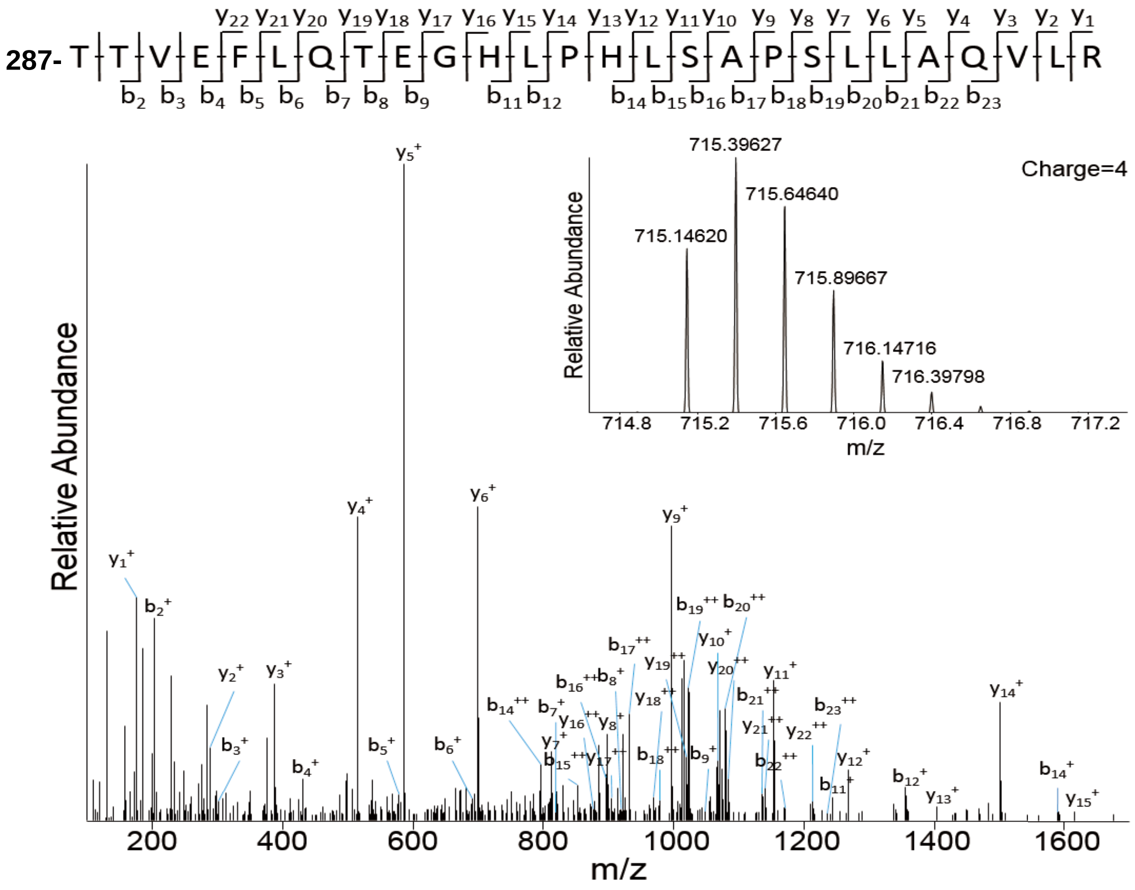

Fig. S2. No modified peptide was identified when D14 without 5DS treatment was subjected to MS/MS analysis.

The unmodified quadruply charged peptide (287-TTVEFLQTEGHLPHLSAPSLLAQVLR-312) of D14 at m/z = 715.39627 was identified when D14 without 5DS treatment was subjected to MS/MS analysis with the same method as mentioned in Fig. 5. Labeled peaks correspond to masses of y and b ions of the peptide displayed on the top, respectively.

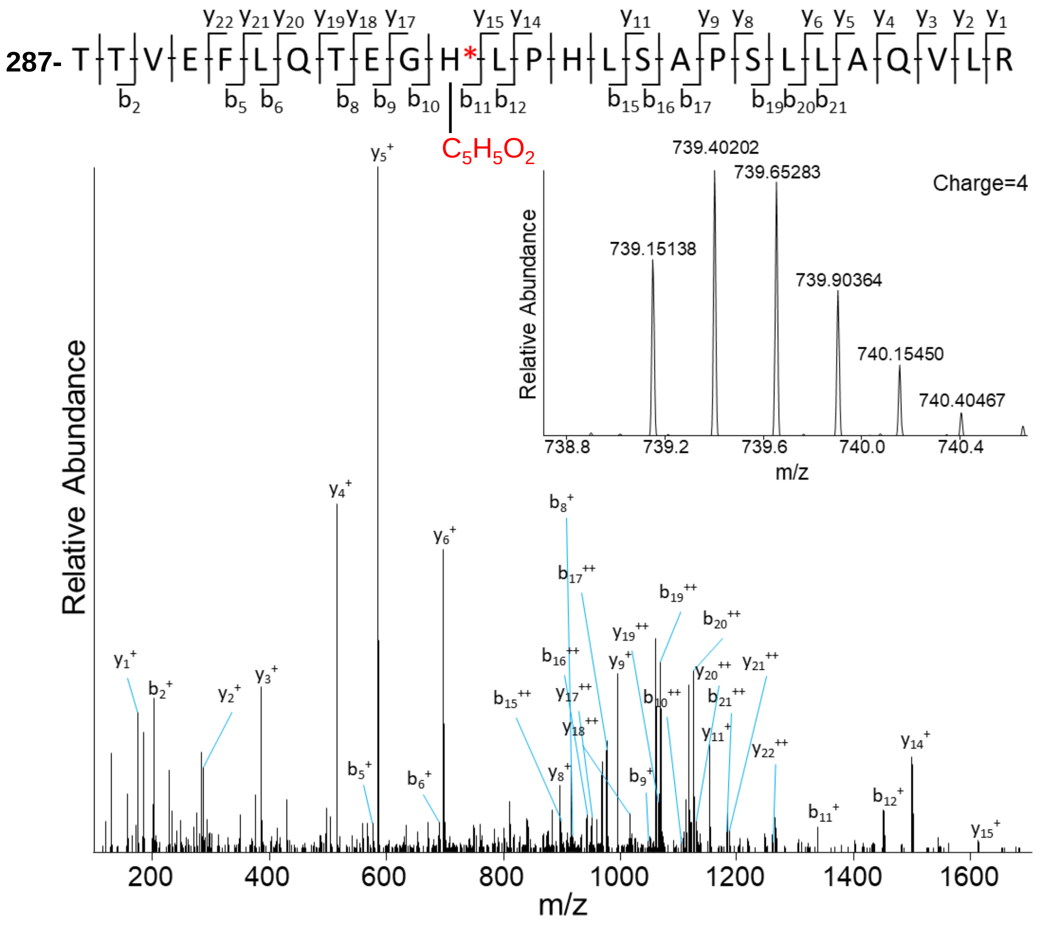

Fig. S3. SL can generate the $C_5H_5O_2$-modification of rice D14 *in planta*.

The existence of CLIM *in vivo* was suggested by successful identification of the same $C_5H_5O_2$-modification on the D14ΔN-Flag protein purified from *Atd14-5 35Spro:D14ΔN* pre-treated with 5DS. The MS/MS analysis was performed with the same method as mentioned in Fig. 5.
